# Loss of Heterozygosity Spectrum Depends on Ploidy Level in Natural Yeast Populations

**DOI:** 10.1093/molbev/msac214

**Published:** 2022-10-07

**Authors:** Abhishek Dutta, Fabien Dutreux, Joseph Schacherer

**Affiliations:** Université de Strasbourg, CNRS, GMGM UMR 7156, Strasbourg 67000, France; Université de Strasbourg, CNRS, GMGM UMR 7156, Strasbourg 67000, France; Université de Strasbourg, CNRS, GMGM UMR 7156, Strasbourg 67000, France; Institut Universitaire de France (IUF), Paris 75005, France

**Keywords:** loss of heterozygosity, *S. cerevisiae*, mitotic recombination, polyploids, genomic instability

## Abstract

The appearance of genomic variations such as loss of heterozygosity (LOH) has a significant impact on phenotypic diversity observed in a population. Recent large-scale yeast population genomic surveys have shown a high frequency of these events in natural isolates and more particularly in polyploids. However, the frequency, extent, and spectrum of LOH in polyploid organisms have never been explored and are poorly characterized to date. Here, we accumulated 5,163 LOH events over 1,875 generations in 76 mutation accumulation (MA) lines comprising nine natural heterozygous diploid, triploid, and tetraploid natural *S. cerevisiae* isolates from different ecological and geographical origins. We found that the rate and spectrum of LOH are variable across ploidy levels. Of the total accumulated LOH events, 8.5%, 21%, and 70.5% of them were found in diploid, triploid, and tetraploid MA lines, respectively. Our results clearly show that the frequency of generated LOH events increases with ploidy level. In fact, the cumulative LOH rates were estimated to be 9.3 × 10^−3^, 2.2 × 10^−2^, and 8.4 × 10^−2^ events per division for diploids, triploids, and tetraploids, respectively. In addition, a clear bias toward the accumulation of interstitial and short LOH tracts is observed in triploids and tetraploids compared with diploids. The variation of the frequency and spectrum of LOH events across ploidy level could be related to the genomic instability, characterizing higher ploidy isolates.

## Introduction

Error-prone DNA recombination and repair can impair fitness but might be counterbalanced by mechanisms that lead to loss of heterozygosity (LOH) (Strathern et al. 1995; Korpanty et al. 2011; Moehring 2011; van Helden 2012; Lancaster et al. 2019). In fact, LOH events can promote the expression of recessive alleles and generate novel allelic combinations, resulting in an increased genetic diversity and the emergence of new genotypes and therefore phenotypes. The mechanisms and evolutionary repercussions of LOH accumulation have been explored in several model organisms, primarily the *Saccharomyces cerevisiae* yeast (Lee et al. 2009; St Charles and Petes 2013; James et al. 2019; Loeillet et al. 2020; Sui et al. 2020; Dutta et al. 2021). LOH events are categorized into interstitial, terminal, and whole-chromosome LOH. Interstitial or short-range LOH events are outcomes of mechanisms leading to gene conversions or double crossovers (Sui et al. 2020). Reciprocal crossovers (RCOs) followed by co-segregation of identical alleles and break-induced replication (BIR) result in terminal or long-range LOH events (Sui et al. 2020). In contrast, in whole-chromosome LOH, one homolog is lost due to chromosomal nondisjunction, alternatively, the remaining chromosome can be duplicated by re-replication. Whole-chromosome LOH is often a downstream consequence of aneuploidy (Pâques and Haber 1999; Llorente et al. 2008; Chapman et al. 2012; Symington et al. 2014).

More recently, mutation accumulation (MA) and serial passaging experiments have been widely employed to elucidate the genome-wide dynamics of LOH accumulation and their phenotypic consequences in different intra- or inter-specific diploid yeast hybrids (Dutta et al. 2017; Heil et al. 2017; Ene et al. 2018; Charron et al. 2019; James et al. 2019; Loeillet et al. 2020; Nguyen et al. 2020; Sui et al. 2020). Globally, LOH events, point mutations, and short indels (<100 bp) were the most frequently detected genetic alterations in yeast diploid genome when the selection is minimal during vegetative propagation (Sharp et al. 2018; Loeillet et al. 2020; Sui et al. 2020). And among them, the LOH event frequency has been estimated to be up to 6 orders of magnitude higher (Dutta et al. 2021). Further exploration even revealed diverse signatures or spectra in distinct genetic backgrounds, each defined by a different pattern of interstitial and terminal LOH events (Loeillet et al. 2020; Nguyen et al. 2020; Dutta et al. 2021). In addition, bursts of genomic instability leading to a large number of LOH events and a complete homozygotization have been highlighted during vegetative progression (Sampaio et al. 2020; Dutta et al. 2021).

While very fruitful, an important limitation of these studies has been the use of laboratory-generated artificial diploid hybrids with uniform levels of heterozygosity (Dutta et al. 2017, 2021; James et al. 2019; Loeillet et al. 2020; Pankajam et al. 2020; Sui et al. 2020). No survey has focused on LOH accumulation and its potential variability in natural isolates showing different ploidy levels. Natural *S. cerevisiae* isolates are mainly diploid, but polyploid isolates (3–5*n*) are frequent and enriched in specific subpopulations such as the beer clade (Gallone et al. 2016; Saada et al. 2022). Additionally, polyploids have been detected in other yeast species (e.g., *C. neoformans* and *C. albicans*) and LOH events are widespread, spanning up to 20% of their genome (Feldmesser et al. 2000; Selmecki et al. 2010; Abbey et al. 2014; Smith and Hickman 2020; Mba et al. 2022).

Here, we therefore conducted a MA experiment to have a better idea of the genome-wide LOH landscape in natural diploid and polyploid *S. cerevisiae* isolates, coming from various ecological and geographical origins. MA lines derived from diploids, triploids, and tetraploids were evolved through 75 single-cell bottlenecks (1,875 generations). Our analysis revealed that the LOH rate and spectrum highly depend on the ploidy level. LOH events occur more frequently in isolates with higher ploidy, with a LOH rate of 9.3 × 10^−3^, 2.2 × 10^−2^, and 8.4 × 10^−2^ events per division for diploids, triploids, and tetraploids, respectively. In fact, polyploids accumulate more internal and shorter LOH tracts.

## Results

### Experimental Design of the Natural MA Lines

Using *S. cerevisiae* as an experimental model, we characterized the genome-wide landscape and dynamics of LOH events in natural diploids as well as polyploid isolates. A total of 76 MA lines were analyzed covering nine different heterozygous genetic backgrounds with three ploidy levels: diploid (*n* = 25), triploid (*n* = 24), and tetraploid (*n* = 27) (table 1; supplementary material figs. S1*A* and *B*and S2, Supplementary Material online). The natural *S. cerevisiae* isolates were chosen from various ecological (e.g., beer, wine, and clinical) and geographical origins (e.g., Africa, South America, and Europe), and designated as ancestors (supplementary material table S1, Supplementary Material online; Peter et al. 2018). Replicate lines were generated from each of the ancestral isolates to set up an MA experiment. The MA lines were propagated purely vegetatively and subjected to single-cell bottlenecks every 48 h for 75 bottlenecks on rich media (YPD) at 30°C. We estimated the MA lines to have undergone an average of approximately 25 divisions per bottleneck for a total of at least 1,875 divisions per line (Liu and Zhang 2019; Dutta et al. 2021). Selection is minimal and genomic changes have accumulated solely due to drift. At the end of the experiment, the genomes of the 76 lines were completely sequenced using a short-read Illumina strategy.

**Table 1.** Natural MA Lines

Ploidy	Std. Name^a^	Het Positions	No. of Sequenced Lines	No. of Bottlenecks
Diploid	BDL	39220	08	75
Diploid	BIE	55580	11	75
Diploid	CBL	56485	07	75
Triploid	BEN	19549	13	75
Triploid	ARN	39985	06	75
Triploid	ATB	60269	05	75
Tetraploid	BEK	26889	09	75
Tetraploid	CBR	53143	08	75
Tetraploid	CKK	77268	11	75

a Standardized names from Peter et al. (2018).

### Variable and Ploidy-Dependent LOH Spectra in *S. cerevisiae*

**Fig. 1.**
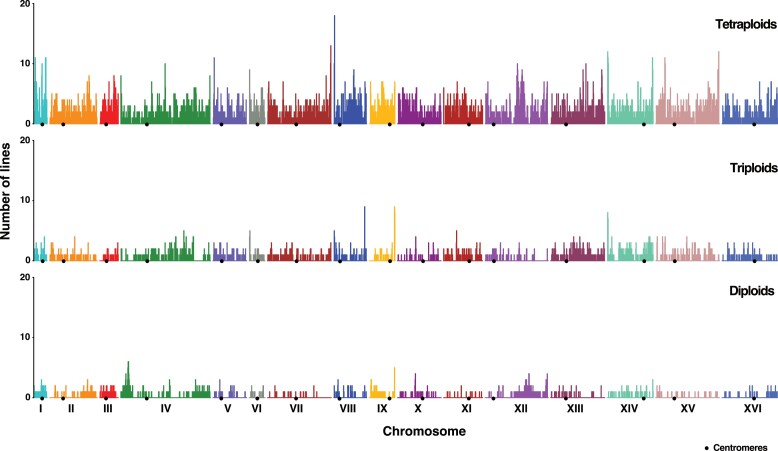
Chromosome-wide map representing cumulative frequencies of regions under LOH in the diploid, triploid, and tetraploid MA lines.

We first identified and determined the LOH events present in the 76 MA lines. LOH events were defined as regions with a length of more than 900 bp and at least three consecutive converted single nucleotide polymorphisms (SNPs) (see Materials and methods). A 900 bp threshold was defined to overcome biases due to the fact that the heterozygosity sites are not evenly distributed across the genome leading to a false positive rate of very short LOH events (supplementary material fig. S3, Supplementary Material online and see Materials and methods). Events greater than 900 bp and supported by at least three consecutive converted SNPs were defined as being under LOH (see Materials and methods; supplementary material fig. S3, Supplementary Material online). Adjacent LOH events were merged if at least two consecutive heterozygous SNPs did not support the disruption. The size of a LOH event corresponds to the distance separating the midpoint between the closest upstream unconverted and converted sites and the midpoint between the closest downstream converted and unconverted sites. From the MA lines data, we compiled a genome-wide LOH map across the three ploidy groups by masking the homozygous regions present in the ancestral isolates (fig. 1).

**Fig. 2.**
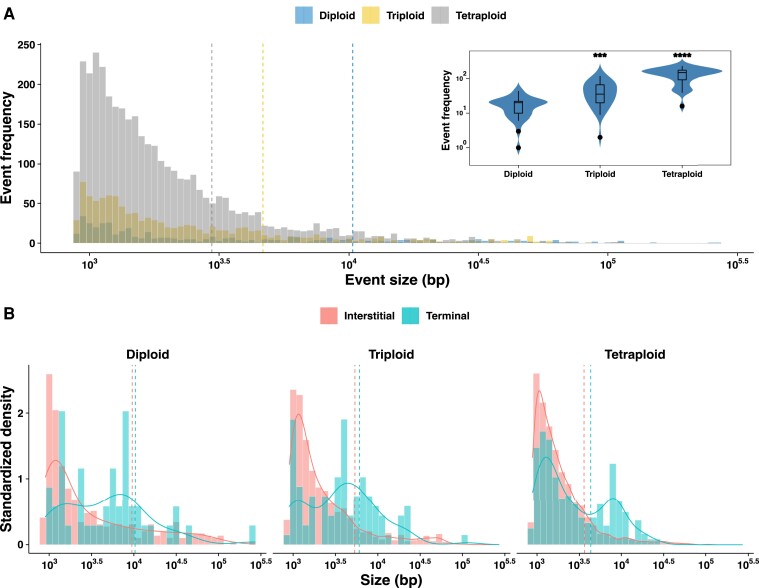
Ploidy-dependent variability in LOH accumulation. (*A*) Overall ploidy wise size distribution of the LOH tracts in the 76 MA lines. The overall average LOH event sizes were **10.3**, **4.6**, and **2.9 kb**, respectively, for the diploids, triploids, and the tetraploids. [*Inset:* Violin plot of the LOH event counts in the MA lines, triploid, and tetraploid lines accumulate significantly greater LOH compared with the diploids (Wilcoxon test, ***P < 0.001; ****P < 0.0001; ns, not significant).] (*B*) Ploidy wise size distribution of the terminal LOH events and not the interstitial events was bimodal. The average tract lengths were **9.9**, **4.5**, **2.9 kb** and **13.3**, **6.6**, **4.3 kb**, respectively, for the interstitial and terminal events.

A total of 5,163 events were identified in the 76 MA lines, 437, 1,087, and 3,639 of which were found in the diploid, triploid, and tetraploid MA lines, respectively (supplementary material table S2 and fig. S4*A*, Supplementary Material online; fig. 2*A*). As previously observed for the artificial laboratory diploids (Dutta et al. 2021), interstitial events were significantly more frequent than terminal events regardless of ploidy level (supplementary material fig. S4*A*, Supplementary Material online). However, the total number of the two types (interstitial and terminal) per line significantly increase with the ploidy (fig. 2*A*; supplementary material table S2, Supplementary Material online). These data also allowed us to estimate the LOH rates and we found a rate of 9.3 × 10^−3^, 2.2 × 10^−2^, and 8.4 × 10^−2^ events per division for the diploids, triploids, and tetraploids, respectively. By determining the rates of interstitial and terminal LOH events, we found the same trend with a ploidy-dependent increase.

Subsequently, we looked at the size distribution of the LOH events. Overall, the size of the LOH events was 10.3, 4.7, and 2.9 kb on average for the diploid, triploid, and tetraploid MA lines, respectively. In fact, we observed a ploidy-dependent decrease in LOH event sizes (fig. 2*A*and*B*; supplementary material fig. S4*B* and table S2, Supplementary Material online). As expected, terminal events were always larger than interstitial events regardless ploidy level (supplementary material fig. S4*B*, Supplementary Material online). Event size variance was consistently high, but highest in the diploids (CV: 245%; Levene’s test, *P* < 2.2 × 10^−16^), followed by the triploids (CV: 213%; Levene’s test, *P* < 2.2 × 10^−16^) and tetraploids (CV: 182%; Levene’s test, *P* < 2.2 × 10^−16^). A chromosome size-dependent increase in LOH events was observed for all ploidy levels but only for interstitial events (supplementary material fig. S5, Supplementary Material online).

Based on the number and size of the events, we estimated the accumulated fraction of the genome under LOH. The acquired genome under LOH was 2.3% on average and ranged from 0.01% to 8%. Polyploid MA lines exhibited a larger fraction of genome under LOH, with 1.8% and 3.3% of the genome in the triploid and tetraploid MA lines, respectively. In comparison, the diploid lines have on average 1.5% of the genome under LOH (supplementary material fig. S6, Supplementary Material online; Wilcoxon test, *P* < 0.01). However, the variance in the accumulated fraction of genome under LOH is higher in diploids (CV: 116%), and significantly lower in triploids (CV: 71%; Levene’s test, *P* < 2.2 × 10^−16^) and tetraploids (CV: 45%; Levene’s test, *P* < 2.2 × 10^−16^), respectively. Nevertheless, the acquired fraction of genome under LOH was much greater (10.5% on average) in the artificial lines we generated in a previous survey (Dutta et al. 2021). Although slightly different, it clearly demonstrates that the accumulated LOH faction is of the same order of magnitude in natural polyploid and diploid isolates.

Overall, the LOH event frequency (interstitial, terminal, and total) was strongly correlated with ploidy level (Spearman’s R = 0.8; *P* < 2.2 × 10^−16^). Only the interstitial event size negatively correlated with ploidy (Spearman’s R = −0.4; *P* < 0.001) while the terminal event sizes were unaffected (Spearman’s R = −0.1; *P* > 0.05) (supplementary material fig. S5, Supplementary Material online). Moreover, the ratio between interstitial and terminal events is similar whatever the level of ploidy (supplementary material fig. S4*C* and table S2, Supplementary Material online) (*P* > 0.05; Kruskal–Wallis test).

### Natural Hybrids Accumulate Less LOH Events Than Artificial Hybrids

We recently explored the accumulation of LOH events in artificial diploid *S. cerevisiae* hybrids generated via crosses between stable haploids, representing different genetic backgrounds (Dutta et al. 2021). We therefore compared the variation of the LOH spectra obtained for natural heterozygous diploid isolates and these artificially generated hybrids. For that purpose, we compared the LOH landscape of three artificial hybrids (BTI/ABA, ACD/AKQ, and ACK/CMQ) having a similar heterozygosity level to that of the natural diploids studied here.

**Fig. 3.**
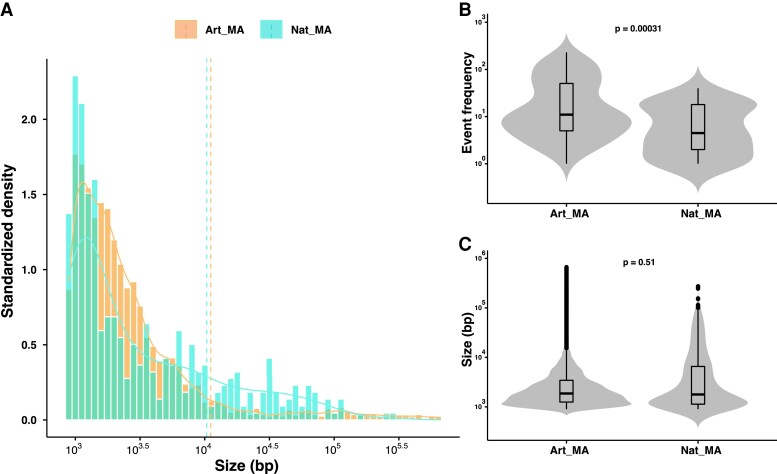
LOH accumulation patterns in artificial versus natural diploids within similar heterozygosity levels. (*A*) The size distribution trends are similar in MA lines derived from artificial (average size—**11.4 kb**) and natural diploids (average size—**10.3 kb**). (*B*) Violin plot of the LOH event counts in artificial versus natural diploid MA lines. Artificial diploid MA lines accumulate significantly greater interstitial and terminal LOH events (Wilcoxon test, *P < 0.05; **P < 0.01; ***P < 0.001; ****P < 0.0001; ns, not significant). (*C*) Violin plot of the LOH event sizes in artificial versus natural diploid MA lines. Interstitial and terminal LOH event sizes are similar (Wilcoxon test, *P < 0.05; **P < 0.01; ***P < 0.001; ****P < 0.0001; ns, not significant).

By comparing these set of MA lines, we found that the size distribution of the LOH events was similar for the artificial and natural lines (fig. 3*A*). The average size of LOH events (including interstitial and terminal events) was 11.1 and 10.3 kb for artificial and natural MA lines, respectively, clearly showing no difference (fig. 3*C*). However, the artificial diploid lines had a significantly higher frequency of events (65.9 events per line on average) than natural diploids (16.8 events per line on average) (Wilcoxon test; *P* < 2 × 10^−16^) (fig. 3*B*). In fact, the LOH rate was 3.2 × 10^−2^ events per division and 9 × 10^−3^ events per division for the artificial and natural lines, respectively. Moreover, both interstitial and terminal event rates were at least 3-fold higher in the artificial lines.

Similarly, the acquired fraction of genome under LOH was far greater in the artificial lines (10.5%) compared with the natural lines (1.5%) (Wilcoxon test, *P* < 0.01). Interestingly, no nearly homozygous lines (>90% of the genome under LOH) were found in the natural lines, while 5% of the artificial lines did, probably highlighting a higher degree of instability in the artificial hybrid genomes.

### Chromosomal Instabilities in the MA Lines

New generated heterozygous and polyploid hybrid genomes generally show a higher rate of genetic instability (Gerstein et al. 2008; Morales and Dujon 2012). Therefore, polyploidy recurrently leads to aneuploidy and genetic diversity, and has been considered a major evolutionary driver. However, the rate of genetic instability in natural diploid and polyploid isolates is not well characterized. Artificially generated diploid and polyploid hybrids have shown to increase or decrease their overall ploidy level during propagation (Charron et al. 2019; Marsit et al. 2021). Moreover, experimentally generated tetraploid *S. cerevisiae* strains frequently revert to a diploid state through loss of chromosomes during mitotic progression (Gerstein et al. 2008; Selmecki et al. 2015). Initially, we assessed the ploidy of all lines using flow cytometry and found no substantial deviations from the ancestral ploidy levels in the MA lines at the end of the experiment. This suggests either that the level of ploidy in the natural isolates is stable, or that recurring bottlenecks in the MA experiment prohibit ploidy changes from being selected, or that the timeframe of the experiment was not long enough to have an impact on that (Sharp et al. 2018; Gerstein and Sharp 2021).

**Fig. 4.**
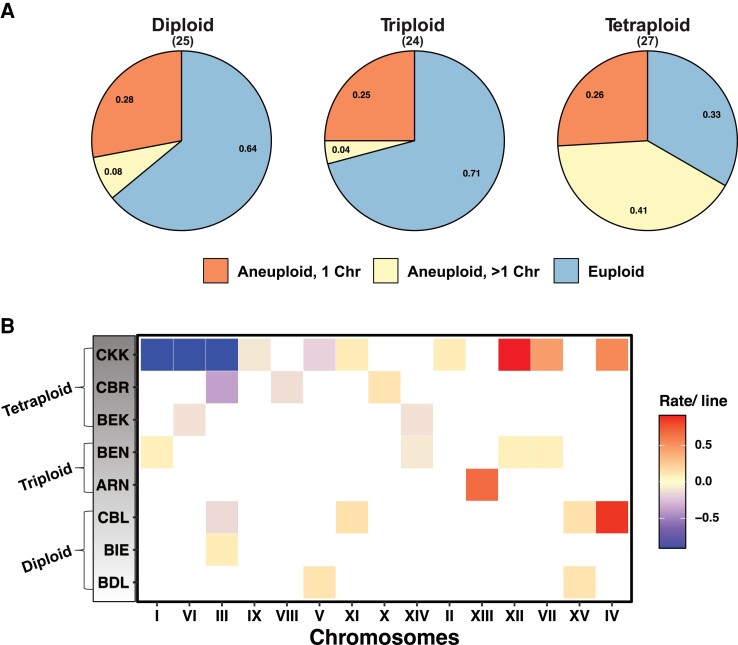
(*A*) Pie chart representing fraction of MA lines euploid, aneuploid (+/− 1 chromosome) or aneuploid (+/− >1 chromosome). (*B*) Frequency of aneuploidies per line across the individual backgrounds. **ATB** (3*n*) derived MA lines and Chromosome XVI overall was never involved in an aneuploidy event and have been excluded from the plot.

We then used sequencing coverage in the MA lines to identify copy number variations, more precisely aneuploidies, resulting from mitotic nondisjunction. In total, we detected 83 chromosomal aneuploidy events in our 76 MA lines (fig. 4*A*and*B*). Aneuploidies were detected in 36% (*n* = 9), 29% (*n* = 7), and 67% (*n* = 18) of the diploid, triploid, and tetraploid lines, respectively (supplementary material table S3, Supplementary Material online). The incidence of aneuploidies was similar in the diploid and triploid lines, as one of the triploid-derived lines (ATB) displayed no aneuploidy, probably indicating a genetic background effect (Hose et al. 2020). Overall, the incidence of aneuploidies was found to be associated with ploidy in the MA experiment (Spearman's R = 0.3; *P* < 2.2 × 10^−16^). The rates of aneuploidies ranged from 1.7 × 10^−4^ to 1.2 × 10^−3^ events per division across ploidy groups, highlighting an 8-fold difference. The rates of chromosomal loss were up to 25-fold higher in tetraploids compared with diploids and triploids. All chromosomes were involved in aneuploidy events, with the exception of chromosome XVI. This is consistent with previous studies with higher frequencies of genomic instability in tetraploids (Gerstein et al. 2008). It should be mentioned that genetic background can influence aneuploidy rates. Indeed, we observed an excess of aneuploidies in specific genetic backgrounds across ploidy groups. For example, most of aneuploidies were detected in CBL (diploid), BEN (triploid), and CBR and CKK (tetraploid). In fact, all the CKK-derived MA lines were ultimately aneuploid (fig. 4*B*). Additionally, we observed no difference in the LOH event rates in the euploid and aneuploid lines (*P* > 0.05, Wilcoxon test).

### Growth Fitness and Fertility of the MA Lines Are Stable Throughout Propagation

**Fig. 5.**
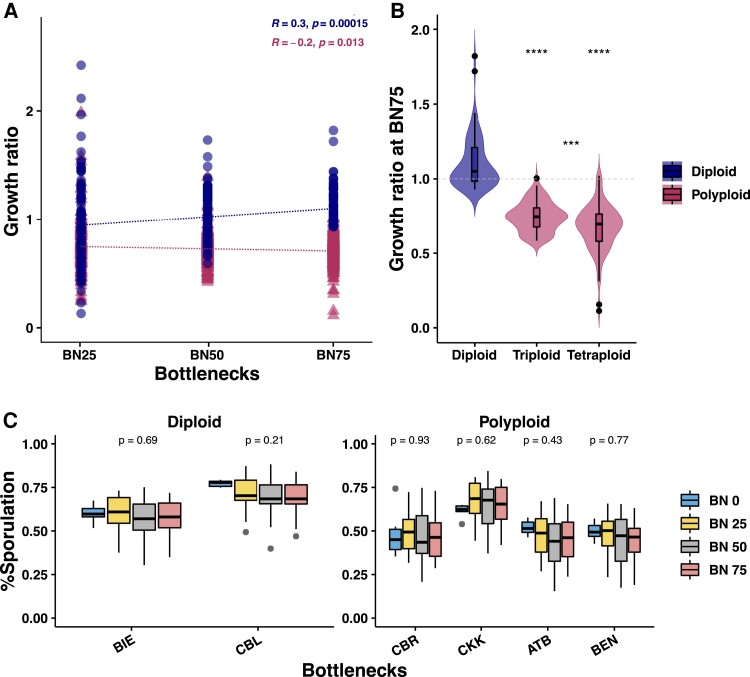
(*A*) Mean growth ratio in the individual MA lines (bottlenecks 25, 50, and 75) compared to the ancestral isolates (bottleneck 0) traced over the course of the experiment. Diploid MA lines display positive growth trend (Spearman’s R = 0.3; *P* < 0.01), while polyploids display a negative growth trend (Spearman’s R = −0.2; *P* = 0.013). (*B*) Violin plot representing growth in the MA lines at the end of the experiment (Wilcoxon test, *P < 0.05; **P < 0.01; ***P < 0.001; ****P < 0.0001; ns, not significant). (*C*) Sporulation efficiencies do not change in the evolved MA lines compared with the ancestral isolates. BDL, ARN, and BEK ancestral isolates as well as the evolved MA lines did not sporulate. Spore viability percentages at bottlenecks 0 and 75 are shown.

We measured the evolution of mitotic growth and fertility of the MA lines over the course of the experiment, i.e., bottlenecks, 25, 50, and 75. Our results show that there are both systematic gain and loss of fitness for the independent diploid lines (supplementary material table S4, Supplementary Material online; fig. 5*A*). Interestingly, both the triploid and tetraploid MA lines never displayed any gain of fitness (fig. 5*A*and*B*; r = −0.2, *P* < 0.05). Globally, we can observe a general gain of fitness in the diploid lines (R = 0.2, *P* < 0.01), while a decline in fitness of the polyploid lines (fig. 5*A*and*B*). Furthermore, the change in fitness was neither significantly associated with LOH nor the aneuploidies.

We then compared the evolution of fertility during the MA lines propagation. We measured both the sporulation efficiency and spore viability components of fertility in these lines. In diploids, meiotic spore fertility negatively correlates with divergence and the gradual restoration can be facilitated by accumulating LOH events (Cubillos et al. 2011; Dutta et al. 2017, 2021). In contrast, evolved tetraploids post whole-genome duplication (WGD) ensured fertility rescue in highly diverged inter-specific *S. cerevisiae*/*S. paradoxus* hybrids (Charron et al. 2019; Marsit et al. 2021). Moreover, in a previous study, we found significant fertility recovery to be associated with accumulation of short interstitial LOH events, as observed in the triploid and polyploid MA lines (Dutta et al. 2021). In the ancestral backgrounds that could sporulate, we tested the sporulation ability of all the derived lines and traced the evolution of the sporulation efficiency for three MA lines, chosen at random. MA lines retained their ability to sporulate, and in fact, we found no significant differences in the evolved lines at the different bottlenecks (supplementary material table S5*A*, Supplementary Material online; fig. 5*C*). Additionally, we measured the meiotic spore fertility of 18 lines across the different genetic backgrounds and ploidy groups at the end of the experiment. Surprisingly, we observed no significant improvements in either the diploid or polyploid MA lines (supplementary material table S5*B*, Supplementary Material online).

## Discussion

The patterns of LOH accumulation in wild *S. cerevisiae* isolates have never been explored and most insights in this regard come from laboratory-generated inter- or intra-specific hybrids. The scale and role of LOH events in hybrid genome stabilization is not well understood in a natural diploid as well as polyploid context. In fact, *S. cerevisiae* and *C. albicans* polyploid isolates have been isolated from diverse ecological and geographical origins, suggesting that they alter their ploidy states to adapt to changing environments (Peter et al. 2018; Todd et al. 2019). Laboratory-generated polyploids display rapid ploidy reductions and chromosomal instabilities in evolution experiments (Gerstein et al. 2008; Selmecki et al. 2015; Gerstein and Sharp 2021). The persistence of polyploids and their evolution is therefore intriguing. Here, we explored the LOH spectrum and chromosomal instabilities in natural heterozygous diploid and polyploid isolates through a MA experiment. Our results demonstrate that polyploids accumulate more LOH events compared with diploids after 1,875 generations. In addition, polyploid MA lines mainly accumulate very short LOH events. Furthermore, we observed that the artificial hybrids exhibit a higher LOH event rate compared with natural isolates. However, the size and distribution of these events are similar to those found in the natural diploid MA lines (Sui et al. 2020; Dutta et al. 2021). Overall, the ploidy level of MA lines generated from wild strains is extremely stable, according to our results. MA polyploid lines showed strong chromosomal instability but the fitness and fertility of these MA lines did not change and is not correlated with LOH or chromosomal instabilities.

Polyploidy itself has been associated with increased genomic instability in yeast (Storchova 2014; Selmecki et al. 2015; Marsit et al. 2021). In fact, polyploid cells in *Drosophila melanogaster* have been shown to display an elevated DNA damage response (Nandakumar et al. 2020). Tetraploid genomes have been shown to be more sensitive to Double Strand Breaks (DSBs) than diploids and, furthermore, heterozygosity is known to hamper the DSB repair process (Strathern et al. 1995; Skoneczna et al. 2015). The high rates of LOH events in polyploid lines may be explained by the fact that heterozygous polyploid genomes may be associated with high DNA damage, which upon recombination repair results in increased LOH events.

A striking observation is the decrease in size of LOH events with increasing ploidy level. The size of these events were 10.3 and 4.3 kb in diploid and polyploid lines, respectively. Similarly, artificial *S. cerevisiae*/*S. paradoxus* hybrid tetraploids that spontaneously evolved from ancestral hybrid diploids in an MA experiment displayed shorter events compared with their diploid counterparts (Marsit et al. 2021). Utilizing a microarray-based approach, Yim et al. (2014) described two distinct classes of conversion events with median sizes of 6 kb (more frequent) and 54 kb (less frequent), arising from different DNA lesions and repair mechanisms. Prior studies have also shown heteroduplex formation, followed by mismatch repair, results in short mitotic conversion events less than 1 kb (Mitchel et al. 2010). The high frequency of LOH events suggests that polyploids undergo more breaks.

Moreover, given the sensitivity of yeast polyploids to DSBs, the involvement of long stretches of DNA in conversions can be deleterious to pairing and disentangling and disrupt ancestral linkage groups. In addition, the size of conversion events is under strong regulation of the MutLβ complex as proposed during meiotic recombination (Duroc et al. 2017). Finally, mutants defective in MMR activity as well as overexpression of MMR proteins have also been proposed to impact the size of conversion events (Campbell et al. 2014; Oke et al. 2014).

We also found that chromosomal instability is higher in polyploids compared with diploids. The rates of aneuploidy were within the range of previous estimates in *S. cerevisiae* diploids, but significantly lower compared with laboratory-generated tetraploids (Gilchrist and Stelkens 2019; Sui et al. 2020; Dutta et al. 2021; Marsit et al. 2021). A recent study has also suggested very high genomic stability and a relatively low incidence of aneuploidies in triploid and tetraploid industrial *S. cerevisiae* isolates post return to growth (RTG) (Mozzachiodi et al. 2021). Increased incidence of aneuploidies compared with diploids may also be a consequence of recombination and multivalent segregation. Interestingly, the patterns of aneuploidies were different in polyploids compared with diploids. Polyploids show more aneuploidies of medium and large chromosomes, and the frequency of chromosome loss is significantly higher. By contrast, we observed aneuploidies to be associated with the small chromosomes and losses to be rare in diploids. This suggests that diploid isolates are more impacted by chromosomal imbalances.

Finally, LOH accumulation and chromosomal instabilities had no effect on the mitotic growth and fertility of the evolved lines. Overall, our results suggest that in natural polyploids, the ploidy level does not vary, probably because the genomic states have been selected for and evolved over a long course of time. Globally, LOH events are key events in terms of genetic diversity for organisms with rare or none outcrossing events such as polyploid yeast isolates. Nevertheless, the phenotypic consequences of this accumulation still need to be assessed.

## Materials and Methods

### Strain Construction and MA Lines Propagation

Table 1 and supplementary material table S1, Supplementary Material online summarize the *S. cerevisiae* strains utilized in this study. Yeast strains were grown on either YPD (yeast extract 1%, peptone 2%, dextrose 2%) or synthetic complete (yeast nitrogen base 0.67%, amino acid mix 0.2%, dextrose 2%) medium at 30°C. Single colonies were isolated from each of the ancestral isolates, and ploidies were confirmed by flow cytometry. These were also checked for their ability to sporulate on 1% potassium acetate-agar. Individual replicate lines were bottlenecked to a single colony every 48 h for at 75 bottlenecks on YPD agar. Intermediates were frozen down every 25 bottlenecks until the end of the experiment. MA lines at the end of the experiment were sequenced. The ploidies of the MA lines were verified via flow cytometry (BD Accuri C6, BD biosciences) before and at end of the MA lines experiment to verify the ploidy level.

### Determining Sporulation Efficiencies and Growth

All MA lines and ancestral isolates were streaked down to single colonies on YPD agar plates. On sporulation media (1% potassium acetate-agar), three separate colonies from each of the lines and ancestral isolates were patched (Argueso et al. 2004). To evaluate sporulation efficiency, 100 cells per sample were counted using an optical microscope at the end of 72 h. The number of dyads, triads, and tetrads divided by the total cell count was used to calculate sporulation efficiency.

Mitotic growth was determined using endpoint colony growth on solid media. MA lines were pre-cultured in YPD broth and pinned onto a solid YPD plate to a 1,536 density format using the replicating ROTOR robot (Singer Instruments). The plates were incubated for 24 h at 30°C and were scanned with a resolution of 600 dpi at 16-bit grayscale. Quantification of the colony size was performed using the R package Gitter and the growth of each of the lines was measured by estimating the growth ratio between the colony size of the MA line at bottlenecks 25, 50, 75 to the respective colony size of the MA line at bottleneck 0 (Wagih and Parts 2014).

### Whole-Genome Sequencing of MA Lines

Genomic DNA was extracted from the MA lines using the Omega yeast DNA kit (Life Science Products). DNA libraries were prepared from 5 ng of total genomic DNA using the NEBNext Ultra II FS DNA Library Kit for Illumina (New England Biolabs) following manufacturers’ protocols. Following quality check using a Bioanalyzer 2100 (Agilent Technologies) and quantification using the Qubit dsDNA HS assay, 4 nM of each library were pooled and run on a NextSeq 500 sequencer with paired-end 150 bp reads by the EMBL Genomics Core Facility (Heidelberg, Germany).

### Read Mapping, Genotyping of Sequencing Data

Sequencing reads from Fastq files were mapped to the masked (RepeatMasker, default parameters, masking simple repeats, and low complexity regions) *S. cerevisiae* R64 reference genome using bwa mem (v0.7.17). The resulting bam files were sorted and indexed using SAMtools (v1.9). Duplicated reads were marked, and sample names were assigned using Picard (v2.18.14). Finally, GATK (v3.7.0) was used to realign remaining reads. Candidate variants were then called using GATK UnifiedGenotyper. The calling was done simultaneously for lines from the same background.

### Analysis of LOH Tracts

After variant calling, SNPs called in each hybrid parental couple were first filtered (bcftools v1.9) to define a set of confident markers expected to be heterozygous in the MA lines. Polymorphic positions in the ancestral strain supported by at least 50 reads were used as markers and filtered based on allele balance (AB) depending on the ploidy: for diploid samples, positions with 0.4 < AB < 0.6 were kept. For triploid and tetraploid samples, positions with 0.2 < AB < 0.8 and 0.05 < AB < 0.95 were kept, respectively.

For each clone, genotyped positions at marker locations with a read depth lower than the mean sequencing depth were filtered out. Fully homozygous positions (0/0 and 1/1, 0/0/0 and 1/1/1, 0/0/0/0 and 1/1/1/1 for diploid, triploid, and tetraploid, respectively) were tagged as LOH, while remaining positions were tagged as heterozygous. Then, heterozygous and LOH tracts were first defined as uninterrupted tracts of successive marker positions with the same tag. Single-marker interruptions between tracts sharing the same tag and within 10 kb of each other were filtered out and subsequent tracts with the same tag were merged. Average LOH tract coordinates were determined as the mean between the coordinates of first or last marker of a given tract and the first previous or next marker around that tract. LOH tracts were tagged as terminal if they overlapped the first or last 20 kb of a chromosome and tagged as interstitial otherwise. Loss of variation events were considered as heterozygous positions. Any tracts with 80% or more overlap and shared by at least 50% of the lines from the same ancestor were excluded. Subsequently, regions under LOH were defined as events >900 bp supported by at least three converted SNPs. The 900 bp threshold was selected by determining, an overlap between the frequencies and sizes of events in the natural MA lines in this study with the artificial diploid MA lines from Dutta et al. (2021), to overcome biases in event detection because of the varying levels of heterozygosity and its distribution (supplementary material figs. S2 and S3, Supplementary Material online). This was also essential as the overall coverage was low 6X–8X and the probability of tracts <900 bp of being false positives were high. The ancestral strains were sequenced with an average coverage of ∼100X (Peter et al. 2018). LOH event rates per line were calculated as *N*/*D*, where *N* is the number of events (Interstitial, Terminal, or total events) and *D* is the total number of divisions/bottlenecks which we assumed to be 25, based on previous literature.

## Supplementary Material

msac214_Supplementary_Data

## Data Availability

All sequenced MA lines listed in supplementary material table S6, Supplementary Material online are available upon request. In addition, sequence data are available from National Centre for Biotechnology Information Sequence Read Archive under accession number: PRJEB45355.
