# Supplementary material for: Loss of Heterozygosity Spectrum Depends on Ploidy Level in Natural Yeast Populations
Source: Mol Biol Evol. 2022 Oct 7;39(11):msac214. doi: 10.1093/molbev/msac214 (PMC9641995; doi:10.1093/molbev/msac214)
Supplement: msac214_Supplementary_Data [file msac214_supplementary_data.zip › Supp_figures.pdf]

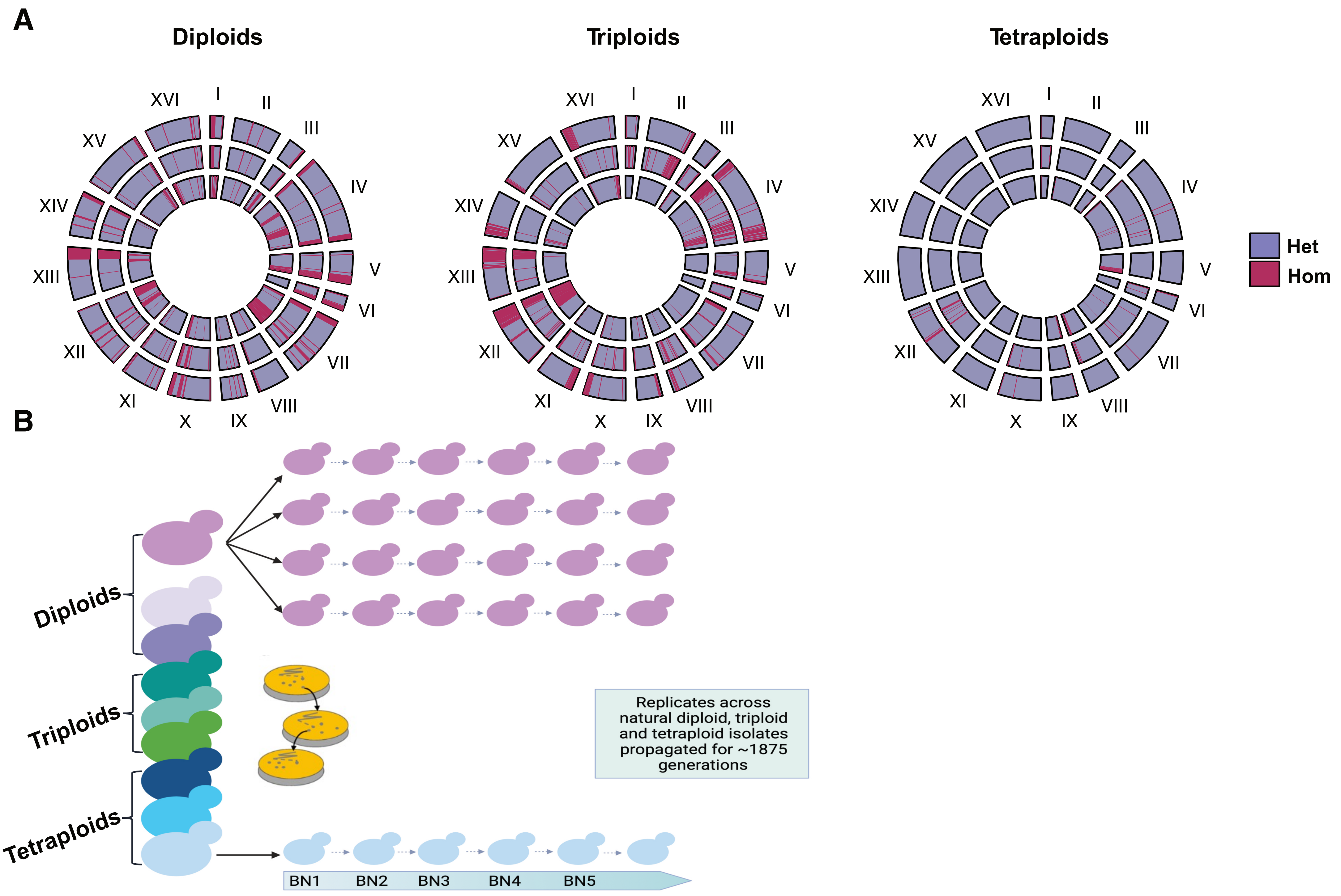
A
Diploids
Triploids
Tetraploids
I
II
III
IV
V
VI
VII
VIII
IX
X
XI
XII
XIII
XIV
XV
XVI
Het
Hom
B
Diploids
Triploids
Tetraploids
Replicates across natural diploid, triploid and tetraploid isolates propagated for ~1875 generations
BN1
BN2
BN3
BN4
BN5

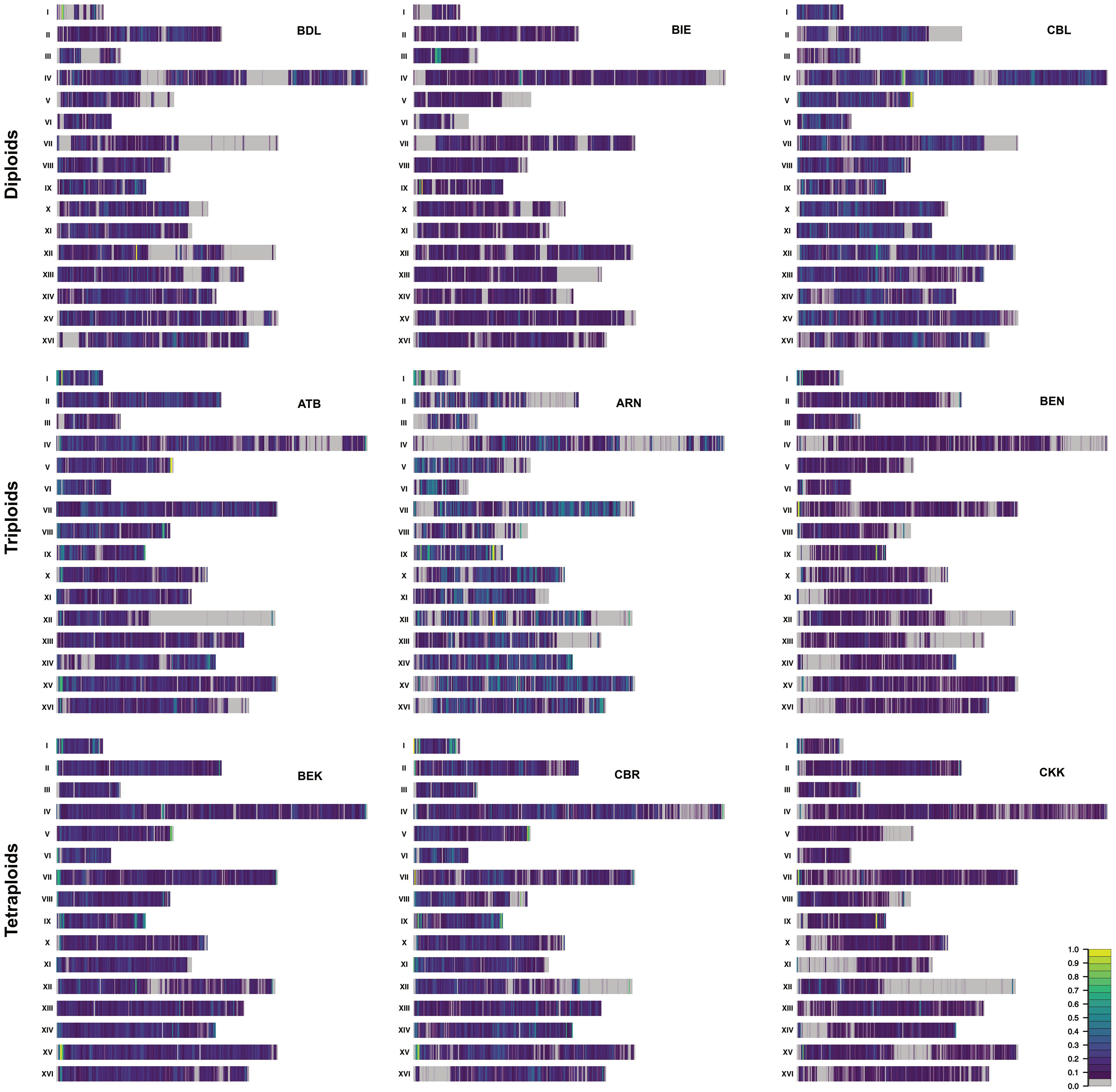

Diploids
Triploids
Tetraploids
BDL
BIE
CBL
ATB
ARN
BEN
BEK
CBR
CKK
I
II
III
IV
V
VI
VII
VIII
IX
X
XI
XII
XIII
XIV
XV
XVI
1.0
0.9
0.8
0.7
0.6
0.5
0.4
0.3
0.2
0.1
0.0

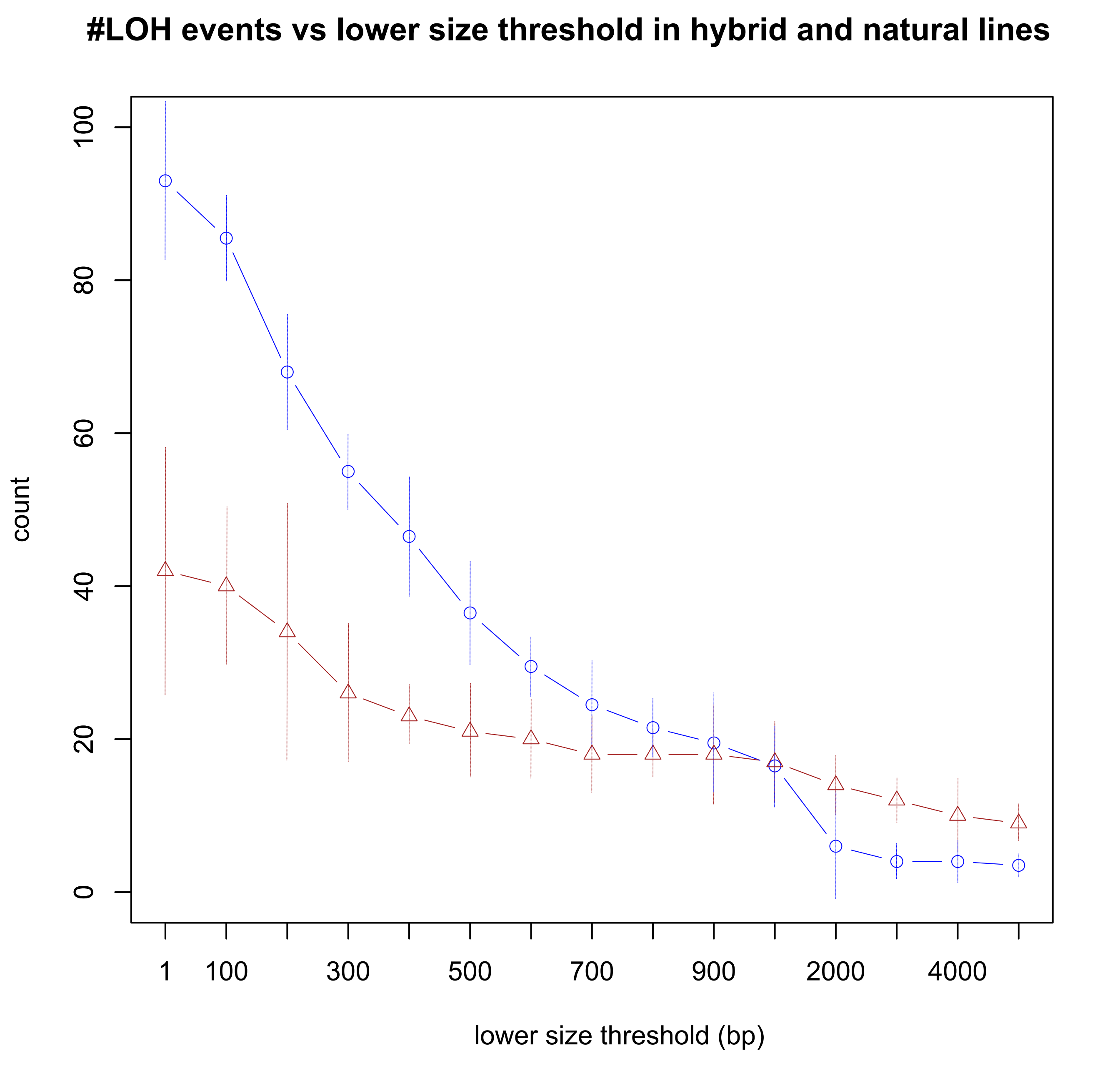

#LOH events vs lower size threshold in hybrid and natural lines
count
0
20
40
60
80
100
1
100
300
500
700
900
2000
4000
lower size threshold (bp)

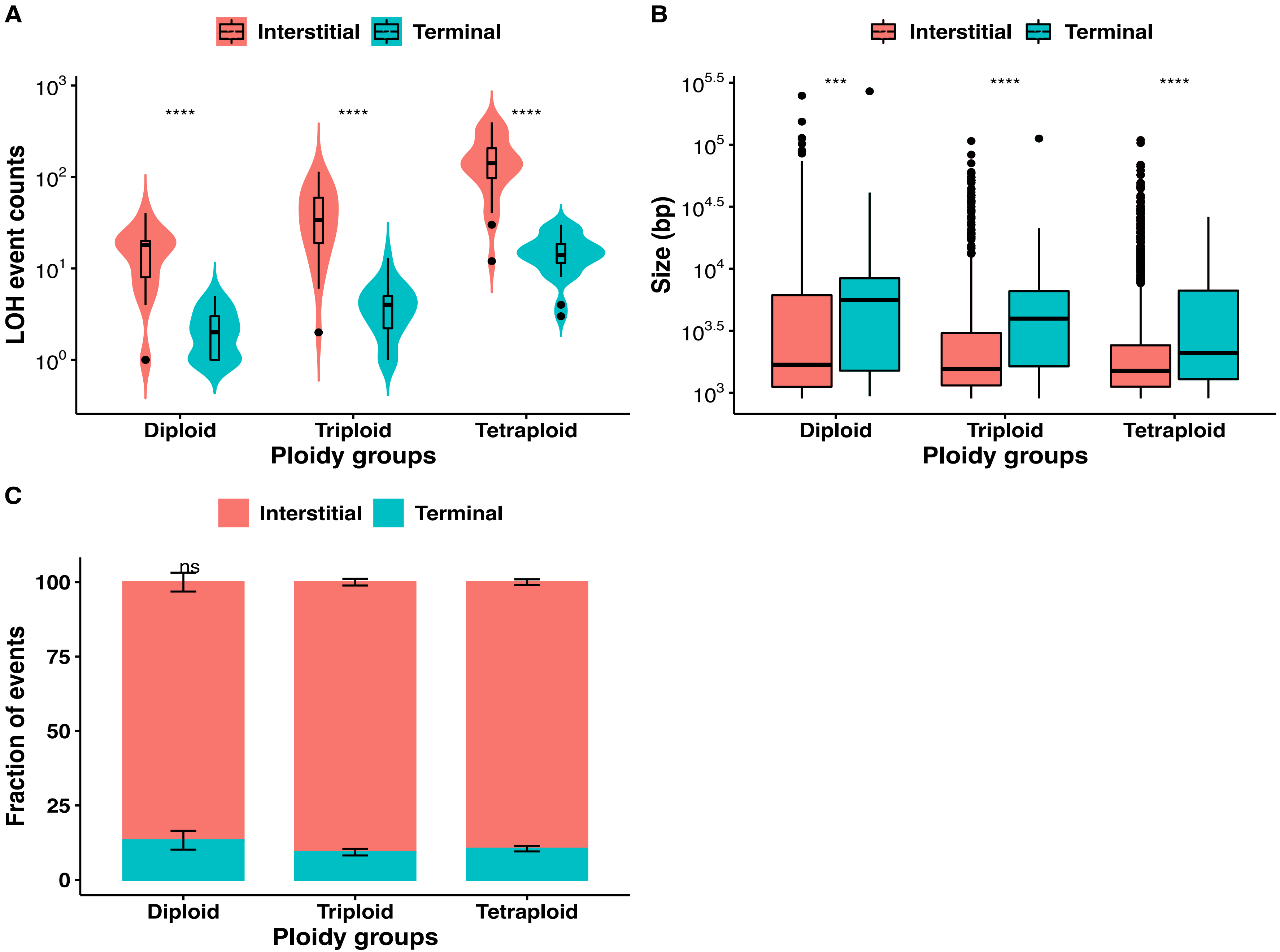
A
Interstitial
Terminal
LOH event counts
10^0
10^1
10^2
10^3
****
****
****
Diploid
Triploid
Tetraploid
Ploidy groups
B
Interstitial
Terminal
Size (bp)
10^3
$10^{3.5}$
10^4
$10^{4.5}$
10^5
$10^{5.5}$
***
****
****
Diploid
Triploid
Tetraploid
Ploidy groups
C
Interstitial
Terminal
Fraction of events
0
25
50
75
100
ns
Diploid
Triploid
Tetraploid
Ploidy groups

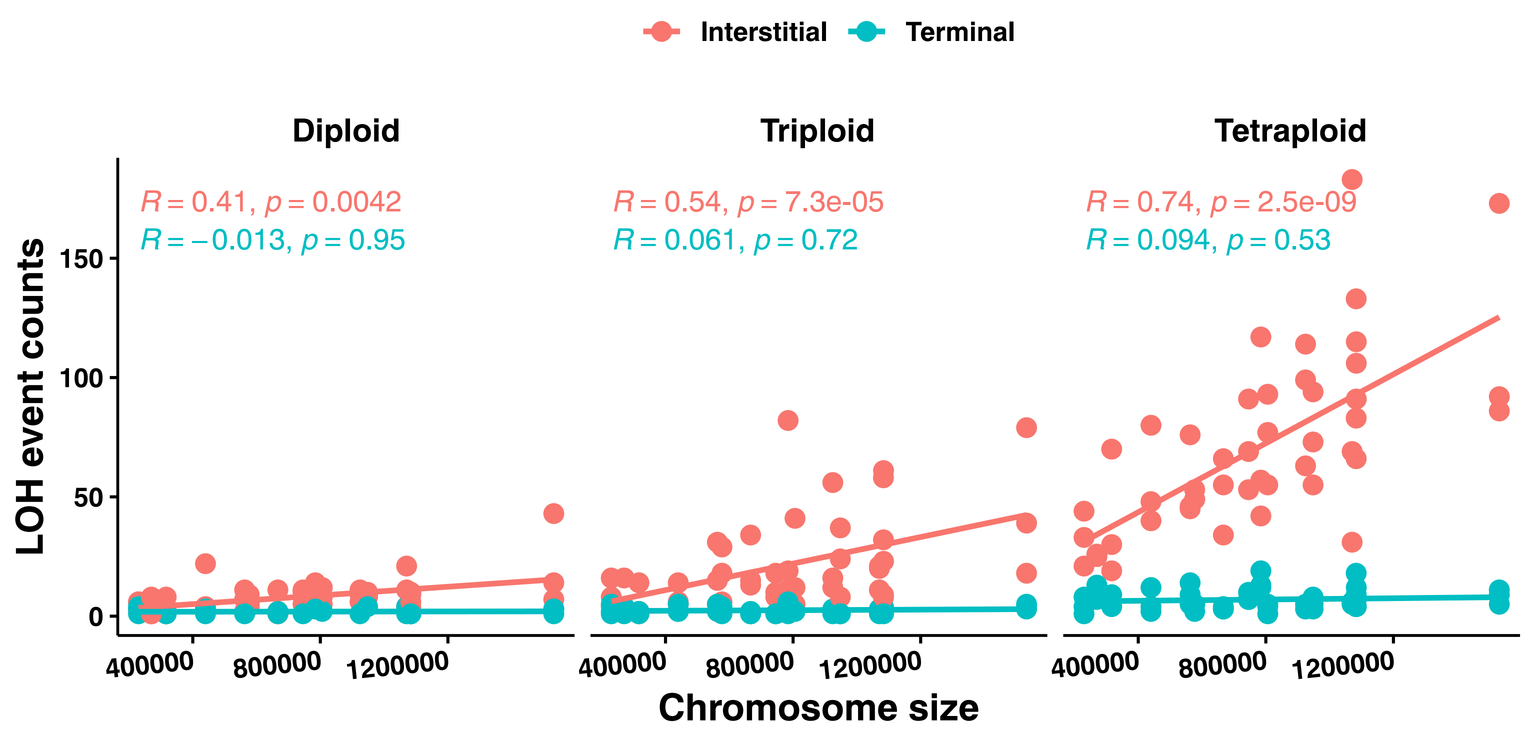

Interstitial
Terminal
Diploid
Triploid
Tetraploid
R = 0.41, p = 0.0042
R = −0.013, p = 0.95
R = 0.54, p = 7.3e-05
R = 0.061, p = 0.72
R = 0.74, p = 2.5e-09
R = 0.094, p = 0.53
LOH event counts
0
50
100
150
400000
800000
1200000
Chromosome size

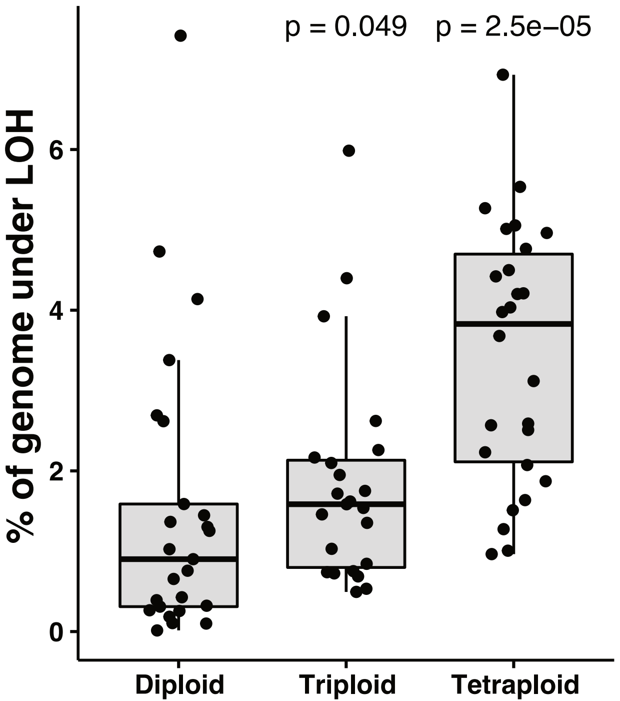

p = 0.049
p = 2.5e−05
% of genome under LOH
0
2
4
6
Diploid
Triploid
Tetraploid
